# Molecular characterization of wheat dwarf virus isolates from Serbia based on complete genome sequences

**DOI:** 10.3389/fmicb.2024.1469453

**Published:** 2024-11-06

**Authors:** Ivana Stanković, Katarina Zečević, Danijela Ristić, Ivan Vučurović, Branka Krstić

**Affiliations:** ^1^Department of Phytopathology, University of Belgrade-Faculty of Agriculture, Institute of Phytomedicine, Belgrade, Serbia; ^2^Department of Plant Diseases, Institute for Plant Protection and Environment, Belgrade, Serbia

**Keywords:** wheat, DAS-ELISA, RT-PCR, genetic diversity, phylogeny

## Abstract

**Introduction:**

Wheat dwarf virus (WDV), the species *Mastrevirus hordei* of the genus *Mastrevirus* in the family *Geminiviridae*, is a cereal virus commonly detected in several European, African and Asian countries that causes economic losses.

**Methods:**

In the spring of 2019, a severe outbreak of wheat dwarfing and yellowing was observed in many winter wheat crops across Serbia. A total of 161 samples were tested for the presence of WDV and other common wheat viruses using double-antibody sandwich enzyme-linked immunosorbent assay (DAS-ELISA). To obtain the complete genome of 23 selected isolates, several overlapping segments of the WDV genome were amplified and sequenced. A phylogenetic tree was constructed using the whole genome sequences of the WDV isolates identified in this study and 40 selected sequences from GenBank.

**Results and discussion:**

The results of DAS-ELISA indicated the presence of WDV in all samples collected from 21 sites in all nine districts surveyed. Further molecular characterization based on complete genome sequencing of 23 selected isolates showed that the Serbian WDV isolates had low nucleotide diversity and were closely related to wheat-infecting isolates from Europe, suggesting the presence of wheat-adapted forms of WDV in Serbia. The constructed phylogenetic tree revealed that Serbian isolates grouped in clade E within the wheat-adapted forms. This study provided the first insight into the genetic structure of WDV in Serbia based on its whole genome sequence. Further studies on the vector biology and population dynamics are needed to better understand the factors influencing the emergence and spread of WDV under local agroecological conditions.

## Introduction

Wheat is a crop that has played a key role in global food security for many centuries (Erenstein et al., 2022). In Serbia, wheat is the second most widely grown cereal after maize,^1^ and the majority of production is grown in the province of Vojvodina (southern parts of the Pannonian Plain), where the climate is particularly favorable for growing winter wheat. More than 50 different viruses are known to infect wheat worldwide, causing severe symptoms with potentially grave impact on yield and quality (Ordon et al., 2009; Liu et al., 2020).

Over the past several decades, the wheat dwarf disease caused by wheat dwarf virus (WDV, *Mastrevirus hordei,* genus *Mastrevirus*, family *Geminiviridae*) has spread more extensively in many cereal-growing areas across Europe, Africa and Asia, causing huge economic losses when its presence reaches epidemic proportions (Ekzayez et al., 2011; Liu et al., 2012; Schubert et al., 2014; Wang et al., 2014; Parizipour et al., 2017; Liu et al., 2020).

Typical symptoms induced by WDV include dwarfing and leaf yellowing, along with reduced headings. The virus may cause yield losses of up to 100% (Jones, 2021), primarily in wheat and barley crops, but it has also been isolated from rye, triticale, and several wild grasses (Lindblad et al., 1999; Köklü et al., 2007; Schubert et al., 2007; Ramsell et al., 2009). It is transmitted by leafhoppers of the genus *Psammotettix* (family *Cicadellidae*) in a circulative, non-propagative manner, with *P. alienus* being considered the most efficient vector in many regions (Lemmetty and Huusela-Veistola, 2005; Wang et al., 2014; Ekzayez et al., 2011). The virus can neither be transmitted by contact between plants, nor by soil, pollen or seeds (Vacke et al., 2004).

Wheat dwarf virus has particles with a characteristic twinned morphology and its genome consists of a monopartite, circular, single-stranded DNA of about 2.6–2.8 kb in length. The WDV genome encodes four proteins, in both the virion- and complementary-sense strands. The virion-sense ORFs encode the capsid protein (CP, gene V1) and the movement protein (MP, gene V2) involved in cell-to-cell movement. The complementary-sense ORFs encode replication-associated proteins (Rep and RepA), expressed from genes C1 and C2. The genome also has long and short intergenic regions (LIR and SIR, respectively), which contain regulatory elements for viral replication and transcription (Dickinson et al., 1996; Kvarnheden et al., 2002; Liu et al., 2014). WDV was initially divided into two main groups: wheat- (WDV-W group) and barley-adapted forms (WDV-B group). Later, phylogenetic analysis identified six strains (A–F) based on sequence similarity between isolates and the phylogenetic relationship; strains A and F originated mainly from barley and were assigned to WDV-B, while strains B–E were assigned to WDV-W specific group (Lindsten and Vacke, 1991; Muhire et al., 2013; Schubert et al., 2014; Wu et al., 2015). Wheat strains show a high level of mutual identity (>98%), while WDV-B isolates are more variable and nucleotide identity among them exceeds 94% (Tóbiás et al., 2011). These two groups of isolates also show differences in their host range: isolates belonging to the wheat-adapted group infect wheat, barley, rye, triticale, and many wild grasses, whereas isolates from the barley-adapted group infect almost exclusively barley and rarely wheat (Köklü et al., 2007; Schubert et al., 2007; Kundu et al., 2009; Wu et al., 2015).

Regardless of the importance of wheat as a crop in Serbia, few studies have been conducted so far to determine the presence of viruses in major cereal-producting areas in our country. Apart from wheat streak mosaic virus (WSMV, *Tritimovirus tritici*), which has been the most frequently isolated virus of wheat in Serbia (Šutić and Tošić, 1964; Vučurović et al., 2020), brome mosaic virus (BMV, *Bromovirus BMV*), as well as barley yellow dwarf virus-PAV and -MAV (BYDV-PAV, *Luteovirus pavhordei* and BYDV-MAV, *Luteovirus mavhordei*) and cereal yellow dwarf virus-RPV (CYDV-RPV, *Polerovirus CYDVRPV*) (Tošić, 1971; Balázs et al., 1990) have also been detected. Interestingly, an investigation conducted by Vučurović et al. (2020) failed to detect the presence of any of these viruses in a substantial percentage of symptomatic wheat samples. In Serbia, WDV was detected for the first time in 2017 (Ristić et al., 2019), but there was later no further information on its occurrence and distribution in our country. Therefore, the aim of this study was to evaluate its distribution in Serbia. Since no sequence data had been available for Serbian WDV isolates, this study also aimed to determine the complete genomic sequences of the isolates collected from different regions in Serbia. The study expands the general knowledge of the molecular diversity and population structure of WDV isolates originating from Serbia and reveals their genetic relationship to those from other parts of the world.

## Materials and methods

### Plant collections and virus detection

Plants with typical dwarfing symptoms were observed in many winter wheat crops across Serbia from March to May of 2019. A total of 161 samples were collected after visual inspection of 21 locations in nine administrative districts of Serbia. Before sampling, disease incidence was estimated in each field by counting plants that exhibits virus-like symptoms in a random batch of 100 plants in four replicates. The sampling method was adjusted to field size. Up to 10 obviously symptomatic plants were selected randomly in small plots (up to 0.5 ha). In contrast, more than 10 symptomatic plants were also collected randomly in large plots (2–3 ha) by moving across the field according to “X model” (Table 1). The samples obtained from leaves of a symptomatic plant were initially tested for WDV and other most common wheat viruses using a double-antibody sandwich (DAS)-ELISA test with commercial polyclonal antisera (Loewe Biochemica, Germany): WSMV, BYDV-PAV and -MAV, BMV, and wheat spindle streak mosaic virus (WSSMV), according to label instructions. Absorbance at 405 nm was measured with an ELISA microplate reader (DASsrl, Italy) and samples showing average optical densities (OD) twice as high as the average of the negative control were considered positive. Commercial positive and negative controls for the viruses mentioned, which are available in commercial kits (Loewe Biochemica, Germany), were included in each ELISA.

**Table 1:** Detection of WDV infection by ELISA test in winter wheat crops of various Serbian districts in 2019 and isolates analyzed in this study.

District	Locations	Wheat variety	Virus detected (No. of analyzed/No. of positive samples)		Isolate selected for full genome sequences	GenBank accession number
			WDV	WSMV		
West Bačka	Odžaci	Apache	3/3	1/3	P-7	PP992758
	Bački Brestovac	Anapurna	2/2	0/2	P-8	PP992759
	Sombor	Apache	2/2	0/2	P-12	PP992760
		Anapurna	11/11	0/11	P-13	PP992761
South Bačka	Bački Petrovac	Anapurna	3/3	1/3	P-20	PP992762
South Banat	Dolovo	Foxyl	2/2	0/2	P-11	PP992763
	Pančevo	Solveig	14/14	0/14	P-36	PP992764
	Vršac	Sobred	3/3	1/3	P-42	PP992765
Central Banat	Sečanj	Farineli	7/7	0/7	P-1	PP992766
Srem	Inđija	Anapurna	4/4	1/4	P-9	PP992767
	Jarkovci	Anapurna	13/13	0/13	P-23	PP992768
	Erdevik	Apache	15/15	0/15	P-29	PP992769
	Šid	Salasar	3/3	0/3	P-30	PP992770
Moravica	Trepča	Apache	8/8	0/8	P-25	PP992771
	Mrčajevci	Apache	5/5	0/5	P-26	PP992772
	Bečanj	Nicol	4/4	0/4	P-27	PP992773
	Čačak	Apache	6/6	0/6	P-28	PP992774
City of Belgrade	Obrenovac	Anapurna	2/2	0/2	P-37	PP992775
Podunavlje	Markovac	Salasar	13/13	0/13	P-31	PP992776
	Velika Plana	Salasar	15/15	0/15	P-32	PP992777
Braničevo	Kostolac	Illico	12/12	0/12	P-34	PP992778
	Požarevac	Illico	14/14	0/14	P-35	PP992779
Total			161/161	4/161		

A total of 22 ELISA-positive samples with a single WDV infection originating from different locations or wheat varieties were randomly selected for molecular characterization based on the complete genome sequence. A previously identified WDV isolate, designated as P1-17 (Ristić et al., 2019), was also included in the study.

### DNA extraction, rolling circle amplification, and PCR

To obtain the complete genome of selected isolates, several overlapping segments of the WDV genome were amplified using a combination of different primers listed in Table 2. Total nucleic acids were isolated from 100 mg of symptomatic leaves using a cetyltrimethylammonium bromide (CTAB) method (Li et al., 2008) and used for PCR amplification, as well as in rolling circle amplification (RCA) to enrich the viral DNA present in the sample. The RCA reaction was performed using the REPLI-g Mini Kit (Qiagen GmbH, Hilden, Germany) according to the manufacturer’s instructions. The RCA product was diluted 1:20 and used for amplification of the target DNA fragments. The PCR amplification was performed in a 25 μL reaction using PCR Master Mix (ThermoFisher Scientific Inc., United States) containing 12.5 μL of Master mix (2x), 2.5 μL of each primer (10 μM), 1 μL of template DNA, and 6.5 μL of molecular-grade water. PCR reactions were performed in an Eppendorf Mastercycler X50l (Eppendorf, Germany) as follows: initial denaturation at 94°C for 2 min, and the final extension at 72°C for 10 min. The annealing temperature and number of cycles are given for each primer pair in Table 2. Negative control was included by replacing template DNA with molecular-grade water, while the Serbian P1-17 WDV isolate was used as a positive control. PCR products were separated by electrophoresis in 1% agarose gel run in 1xTBE buffer and stained with ethidium bromide.

**Table 2:** Primers used for amplification and sequencing.

Primer	Sequence (5′–3′)	The primers positionin the genom sequences	Cycling (temperature/time)				Amplicon size (bp)
			Denaturation	Annealing	Extension	No. of cycles	
V1Fr	CGGCTTTTCGTGAGTGCGC	30–48	94°C/20 s	55°C/30 s	72°C/60 s	30	802
V2Rev2	GGCATCGTAAAGATGTCAGTGG	810–831					
735F	TCCGTTCATCGGTCCAGTCCG	735–756	94°C/60 s	55°C/60 s	72°C/60 s	35	1,152
1886R	ACTCCGTAAGCCTCGAATCC	1,866–1,886					
WD1	GTGAACTTGAATGGAATGTC	1,611–1,630	94°C/20 s	55°C/30 s	72°C/60 s	30	1,287
WD2	GCGCACTCACGAAAAGCCG	30–48					
WD9	CGGCAGGTCCTTAGCGAAA	2,517–2,535	94°C/20 s	55°C/30 s	72°C/60 s	30	504
WD10	ACACAAAKGTYCGCCAGGC	253–271					

### Sequence analysis and construction of phylogenetic trees

Products of an expected size, obtained in PCR assays were purified with a QIAquick PCR Purification Kit (Qiagen) and at least three samples of each amplicon were sent for sequencing in both directions on an automated sequencer (Macrogen-Europe BV) using the same primers as in amplifications. For each isolate, sequence data were analyzed and assembled using the ClustalW program (Thompson et al., 1994) implemented in MEGA X software (Kumar et al., 2018), and complete sequences of the WDV genome of isolates were obtained. All sequences generated in this study were deposited in the GenBank database and assigned accession numbers (Table 1). The obtained sequences were compared with each other by calculating nucleotide identities (nt) using the p-distance model implemented in MEGA X software (Kumar et al., 2018). All Serbian sequences were also compared with previously reported isolates available in the GenBank using BLAST algorithm.^2^

A phylogenetic tree was inferred using the maximum parsimony method implemented in MEGA X. The bootstrap method (1,000 replicates) was used to assess the reliability of individual nodes in the phylogenetic tree, and bootstrap values <70% were omitted. The sequence AM296025 of a German isolate of oat dwarf virus (ODV) was used as outgroup sequence. The phylogenetic trees were visualized using iTOL v6 (Letunic and Bork, 2024). Intra- and intergroup diversity was calculated using Diversity Estimation analysis and K2 + G evolutionary model, both implemented in MEGA X.

### Recombination analysis

Recombination analysis was carried out using the Recombination Detection Program v.4.16 (RDP4). Recombination events and recombination break points were analyzed using the RDP, GENECONV, BootScan, MaxChi, Chimera, SiScan, and 3Seq methods implemented in the RDP4 program (Martin et al., 2015). Default parameter values were used, and a sequence was considered recombinant if the recombination signal was supported by at least five methods with *p* values ≤0.05.

## Results

### Symptoms and virus detection using DAS-ELISA

**Figure 1:**
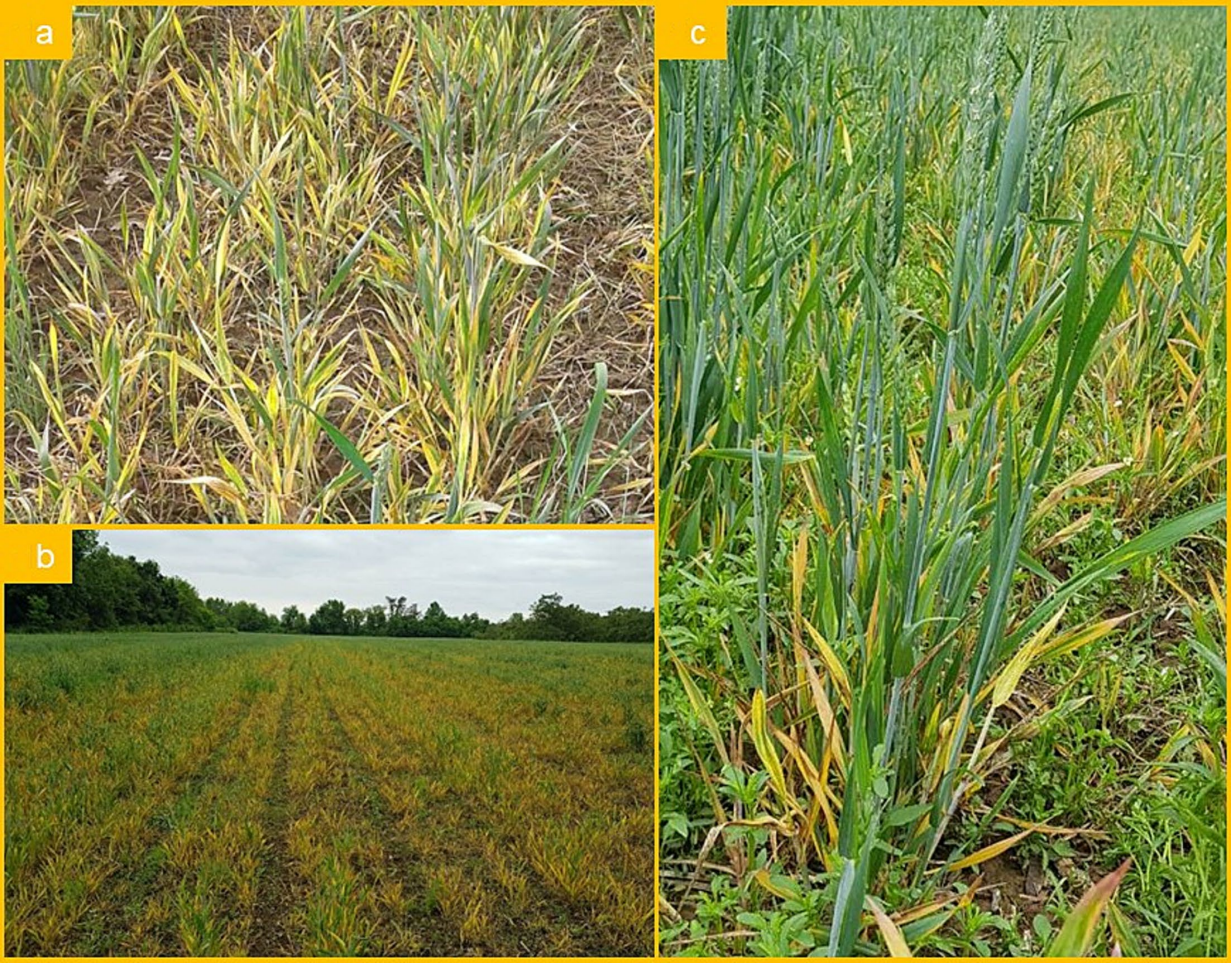
Symptoms of wheat dwarf (WDV) infection on wheat: **(A)** yellowing of leaf; **(B)** severe yellowing and plant dwarfism; **(C)** yellowing, necrosis, and reduced heading formation.

During visual inspection of winter wheat fields of different varieties over the period from March to May 2019, symptoms resembling those caused by WDV were observed at various locations throughout Serbia. The first symptoms included various types of leaf yellowing (Figure 1A) and necrotic leaf spots, as well as different degrees of dwarfing (Figure 1B), and reduced heading (Figure 1C). Later, these plants decayed due to necrosis and there were large yield losses in many locations regardless of wheat variety. The average estimated disease incidence mostly ranged between 40 and 60%, but it was extremely high (up to 100%) in some fields.

**Figure 2:**
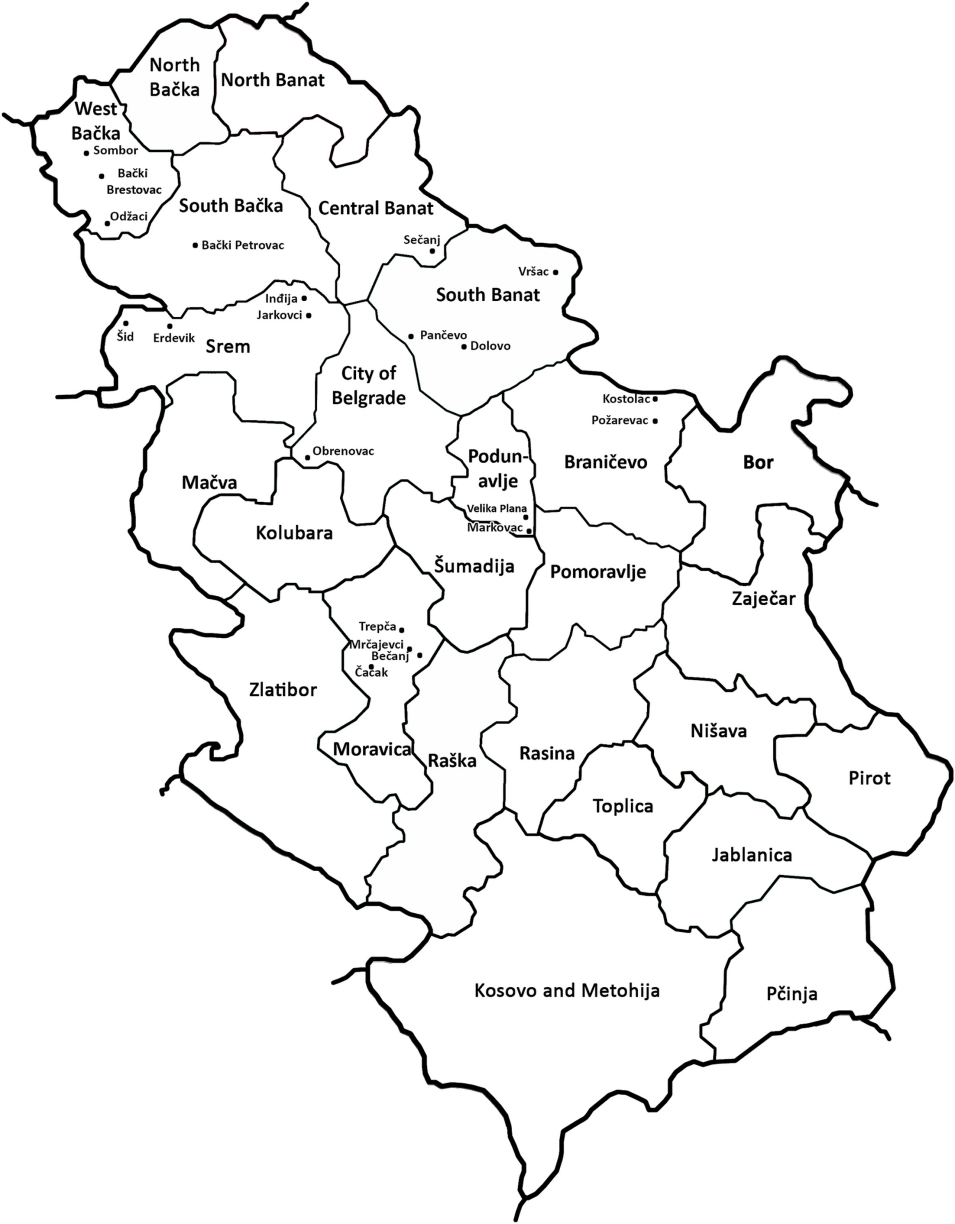
Locations with wheat dwarf virus (WDV) infection in Serbia in the spring of 2019.

The results of DAS-ELISA test revealed that WDV was present in all analyzed samples collected at 21 locations in all of the nine inspected districts (Table 1; Figure 2), indicating that WDV was common and widely distributed throughout Serbia. In a single infection, WDV was detected in 97.52% of the samples tested. The presence of WSMV was proved in 2.48% of the samples tested at four locations and the virus was in mixed infection with WDV. No other viruses were detected.

### Genetic diversity of Serbian WDV isolates

All 22 selected isolates collected during this study, as well as the previously identified isolate P1-17 (PP992780), were fully sequenced and submitted to GenBank. Details of the origin of selected samples and accession numbers in GenBank are given in Table 1. Analyses of the WDV complete genome sequence showed high levels of identity among the Serbian WDV isolates originating from wheat, which exhibited nt identities ranging from 98.3 to 99.9%. Isolates P-26 (Mrčajevci) and P-31 (Markovac) showed the highest percentage of nt identity with isolates P-32 (Velika Plana) (99.9%), while P-7 (Odžaci) was the most distant from isolates P-12 (Sombor) (98.3%). Also, isolate P-7 was the most distant from all other Serbian WDV isolates and shared 98.3–99.4% nt identity with theme. BLAST results revealed that Serbian WDV isolates shared the highest nt identity of 98.95–99.78% with those available in GenBank. Most Serbian isolates showed the highest nt identity of 98.95–99.78% with the Ukraine isolate FN806784. Isolates P-7 shared the highest nt identity of 99.75% with the Czech isolate FJ546188, while the sequence of isolate P-12 had the highest nucleotide identity of 99.42% with one isolate each from the Czech Republic and Germany (FJ546191 and AM296023, respectively).

It is noteworthy that none of the sampled plants showed evidence of mixed infections with different WDV isolates characterized by multiple peaks in the sequencing ladder, and none of the seven recombination detection programs showed possible recombination events among the examined WDV isolates.

### WDV phylogenetic analysis

**Figure 3:**
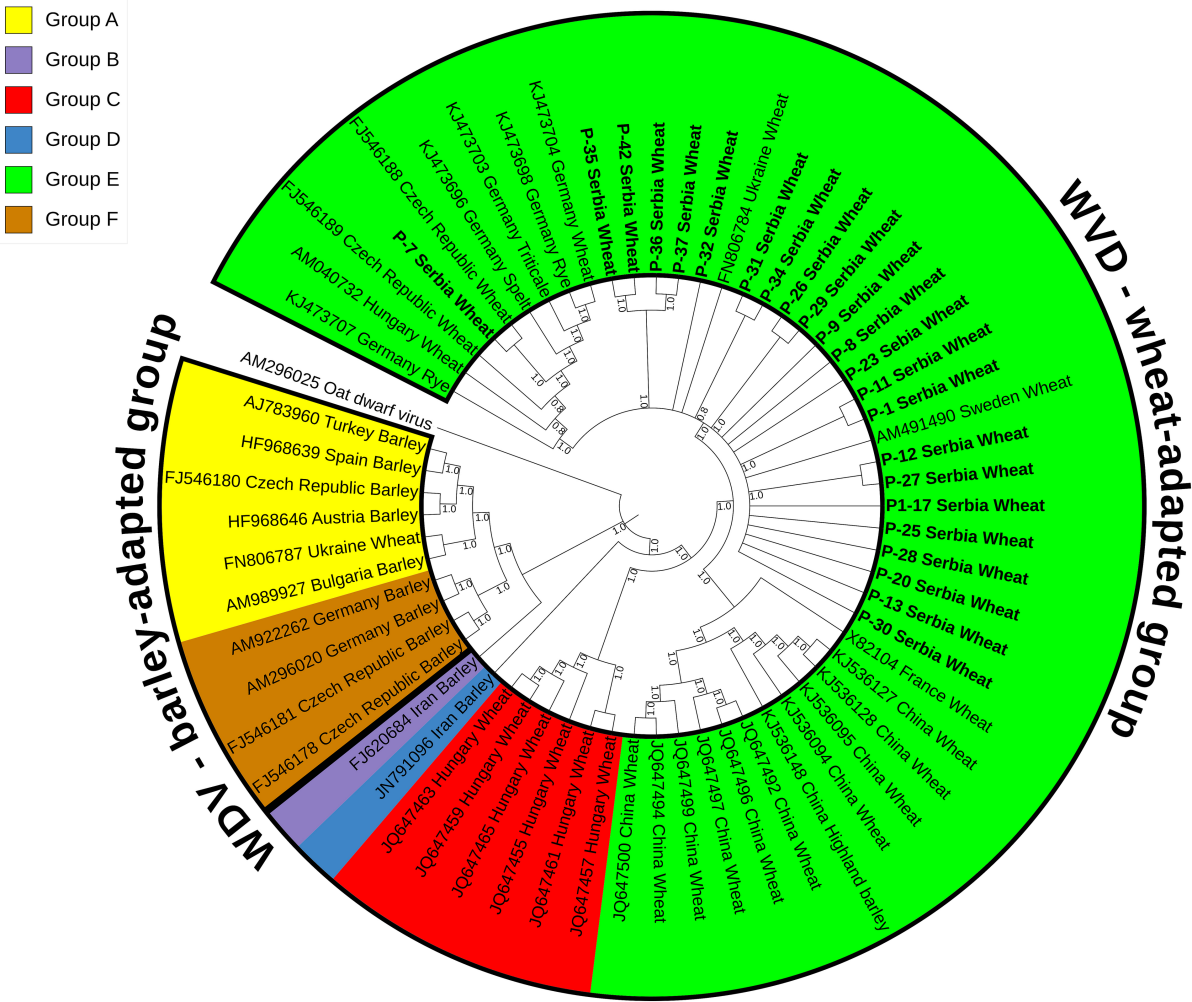
Maximum parsimony tree based on 63 complete sequences of wheat dwarf virus (WDV) isolates constructed using MEGA X and bootstrap analysis with 1,000 replicates. Bootstrap values of 70% and above are shown at nodes. Clade A–F were labeled in different colors in the tree. The isolates from this study are bolded and underlined.

A maximum parsimony tree (Figure 3) was constructed using the whole genome sequences of the WDV isolates identified in this study and selected sequences of 40 previously characterized isolates retrieved from GenBank (Supplementary Table S1). The isolates were divided into two groups, wheat- and barley-adapted, with a genetic diversity between them of 0.197 ± 0.010. The barley-adapted group (0.039 ± 0.003) comprises clades A and F, while clades B, C, D, and E belong to the wheat-adapted group (0.032 ± 0.002). Genetic diversity among the six clades of WDV isolates ranged from 0.049 ± 0.004 to 0.209 ± 0.011, while the diversity within each clade was: 0.036 ± 0.003 (A), 0.009 ± 0.001 (C), 0.036 ± 0.003 (E), and 0.007 ± 0.001 (F). European isolates originating from barley were grouped into clades A and F. Clade C comprised wheat isolates from Hungary, clades D and B contained a barley isolate each from Iran, whereas clade E consisted of European and Chinese isolates from different hosts, including wheat. The 23 Serbian WDV isolates were grouped into clade E, together with other European WDV isolates manly from wheat. However, the sequence of isolate P-7 showed some diversity and belonged to a different subgroup within clade E, showing a closer phylogenetic relationship with the Czech isolate CZ1561 than with other Serbian WDV isolates.

## Discussion

More than 50 different viruses are known to occur in wheat (Ordon et al., 2009), including WDV as the causal agent of wheat dwarf disease. Many studies have shown that WDV is the most commonly detected and widely distributed virus infecting cereals in many European countries (Áy et al., 2008; Kundu et al., 2009; Tóbiás et al., 2011; Schubert et al., 2014; Trzmiel, 2018). The increased WDV distribution and occurrence in areas where the virus had not been previously reported are attributed to changes in agricultural practices, global climate change and the use of cereal varieties with high susceptibility to WDV (Pfrieme et al., 2023). Accordingly, WDV had been expected to appear in Serbia and, indeed, the virus was detected for the first time in 2017 (Ristić et al., 2019).

After this single record, the virus presence reached epidemic proportions 2 years later, in 2019, when symptoms of yellowing and severe dwarfism (Figure 1) were observed in many winter wheat crops across Serbia. To confirm the presence of WDV and determine the possibility of mixed infection, all samples were serologically analyzed for the presence of five economically important wheat viruses. Serological analyses of wheat samples revealed the presence of WDV in 100% of tested samples collected at 21 locations in nine districts, indicating that WDV is a widespread and prevalent wheat virus in our country. In addition to this virus, WSMV was also detected at a much lower frequency and always in mixed infections with WDV (Table 1). Many other studies (Kundu et al., 2009; Tóbiás et al., 2011; Schubert et al., 2014) have also shown that WDV is one of the most dangerous and frequently identified cereal viruses in different parts of Europe. WDV is considered one of the most widespread wheat viruses, causing major economic damage in Hungary (Áy et al., 2008). In Poland, WDV is a serious problem in the production of various cereals, including triticale, wheat, barley, and rye (Trzmiel, 2018). In Iran, WDV epidemics also occur regularly in various cereal crops as well as in several grassy weeds (Parizipour et al., 2017).

Analyses of the complete genome sequences of 23 viral isolates originating from different locations or wheat varieties (Table 1; Figure 2) allowed to present the first molecular characterization of WDV from Serbia. The sequence diversity established in this study showed that the Serbian WDV isolates were highly homologous to each other regardless geographic origin and to the corresponding sequences of wheat-infecting WDV isolates from other parts of the world, indicating the presence of wheat-adapted forms of WDV in Serbia. Similar low genetic variation was also observed in WDV-wheat strains from Germany (Schubert et al., 2007), the Czech Republic (Kundu et al., 2009), Sweden (Ramsell et al., 2008), and Ukraine (Tóbiás et al., 2011). This is not surprising given that it was previously shown that wheat isolates had at least 98.3 to 98.8% sequence similarity with the respective strains, whereas barley WDV isolates had at least 94% similarity (Pfrieme et al., 2023). However, isolate P-7 showed a closer relationship to the Czech isolate CZ1561 than to other Serbian WDV isolates, especially when compared to isolate P-8 collected at the same location, indicating that the virus might have been introduced more than once into Serbia or might have been altered in some cases by the arrival of new WDV isolates.

Phylogenetic analyses (Figure 3) based on the whole genome sequences revealed two distinct groups, the wheat- and the barley-adapted, consistent with previous phylogenetic studies (Tóbiás et al., 2011; Muhire et al., 2013; Wu et al., 2015; Mishchenko et al., 2022). The Serbian WDV sequences clustered in clade E within the wheat-adapted group, suggesting a possible common origin, but some separate groupings of isolate P-7 showed a degree of genomic variation. This finding is interesting considering that the phylogenetic relationships of WDV isolates are usually highly dependent on the geographic origin of the virus (Köklü et al., 2007). However, as noted by Tóbiás et al. (2011), the occurrence and behavior of vectors, plant varieties grown in any given area, agricultural practices and, most importantly, the original source of the virus in a country should also be considered when assessing the spreading, as well as evolutionary divergence of WDV.

Even though natural infection with both strains, WDV-W and WDV-B, has been reported in wheat (Tóbiás et al., 2011), we did not detect the WDV-B strain in Serbia. Moreover, while symptoms on wheat are severe and intense, no symptoms have been observed on barley. In addition, none of the seven recombination detection programs showed any possible recombination events among the WDV isolates examined, although several new types of recombinants have been identified between wheat and barley strains and within autologous groups (Wu et al., 2015; Parizipour et al., 2017).

Symptoms typical of WDV, high WDV frequency, significantly low frequency of mixed infection with WSMV, and absence of other viruses, including those that also cause dwarfism, converged toward a conclusion that the causal agent of the disease that appeared in extremely high infection rates in wheat crops in Serbia in 2019 was WDV. After the 2019 epidemic, no symptoms of yellowing and dwarfing of wheat were seen and WDV was not detected in field surveys of winter wheat in Serbia for 4 years (unpublished data). A similar situation was reported in many other countries, including Sweden (Lindblad and Waern, 2002), Austria (Schubert et al., 2014) and Hungary (Tóbiás et al., 2011). WDV incidence in wheat and other cereals has been reported to differ greatly from year to year and even field to field (Manurung et al., 2004; Širlova et al., 2005).

Although the determination of the vectors present was not performed in this study, it is already known that the vector, *P. alienus*, is present and widespread in our country (Cvrković et al., 2010; Jović et al., 2010; Jakovljević et al., 2013). It is assumed that epidemics are triggered by the use of cereal varieties with high susceptibility to WDV in combination with favorable conditions that lead to high vector incidence. However, further studies on the population density and activity of leafhoppers under local agroecological conditions are needed to develop appropriate measures and improve the ability to control the virus and its vectors to minimize the risks of WDV epidemics and crop losses, as resistant wheat and barley varieties are not yet available.

## Data Availability

The datasets presented in this study can be found in online repositories. The names of the repository/repositories and accession number(s) can be found in the article/Supplementary material.

## Footnotes

^1^ www.stat.gov.rs

^2^ http://www.ncbi.nlm.nih.gov/BLAST/
