# Supplementary material for: Molecular characterization of wheat dwarf virus isolates from Serbia based on complete genome sequences
Source: Front Microbiol. 2024 Nov 6;15:1469453. doi: 10.3389/fmicb.2024.1469453 (PMC11576430; doi:10.3389/fmicb.2024.1469453)
Supplement: Supplementary file 1 [file Table_1.docx]

**Supplementary Table S1.** Sequences of wheat dwarf virus (WDV) isolates used in the phylogenetic analysis

| **Virus strain [isolate]** | **Country** | **Source** | **GenBank Accession number** |
| --- | --- | --- | --- |
| WDV-W [Spelt9] | Germany | *Triticum spelta* | KJ473696 |
| WDV-W [Winter_rye_101] | Germany | *Secale cereale* | KJ473698 |
| WDV-W [Triticale_118] | Germany | *x Triticosecale* | KJ473703 |
| WDV-W [Winter_wheat_120] | Germany | *Triticum aestivum* | KJ473704 |
| WDV-W [Rye9_3] | Germany | *S. cereale* | KJ473707 |
| WDV-B [McP20] | Germany | *Hordeum vulgare* | AM296020 |
| WDV-B [SA12EcoFL2] | Germany | *H. vulgare* | AM922262 |
| WDV-B [CZ6482] | Czech Republic | *H. vulgare* | FJ546178 |
| WDV-B [CZ11105] | Czech Republic | *H. vulgare* | FJ546180 |
| WDV-B [CZ11229] | Czech Republic | *H. vulgare* | FJ546181 |
| WDV-W [CZ1561] | Czech Republic | *T. aestivum* | FJ546188 |
| WDV-W [CZ1561] | Czech Republic | *T. aestivum* | FJ546189 |
| WDV-W [WDV-B] | Hungary | *T. aestivum* | AM040732 |
| WDV-W [HUNGARY-kp10-1] | Hungary | *T. aestivum* | JQ647455 |
| WDV-W [HUNGARY-kp10-4] | Hungary | *T. aestivum* | JQ647457 |
| WDV-W [HUNGARY-kp10-6] | Hungary | *T. aestivum* | JQ647459 |
| WDV-W [HUNGARY-kp10-9] | Hungary | *T. aestivum* | JQ647461 |
| WDV-W [HUNGARY-kp10-11] | Hungary | *T. aestivum* | JQ647463 |
| WDV-W [HUNGARY-kp10-15] | Hungary | *T. aestivum* | JQ647465 |
| WDV-W [Enkoping2] | Sweden | *T. aestivum* | AM491490 |
| WDV-W [French] | France | *T. aestivum* | X82104 |
| WDV-B [Bg17] | Bulgaria | *H. vulgare* | AM989927 |
| WDV-B [ES1BDV] | Spain | *H. vulgare* | HF968639 |
| WDV-W [WDV-Uk-Miron] | Ukraine | *T. aestivum* | FN806784 |
| WDV-B [WDV-Uk-Odessa] | Ukraine | *T. aestivum* | FN806787 |
| WDV-B [Barley] | Turkey | *H. vulgare* | AJ783960 |
| WDV-B [AU196BDV] | Austria | *H. vulgare* | HF968646 |
| WDV-W [HNZZ08-6] | China | *T. aestivum* | KJ536094 |
| WDV-W [HNZZ08-7] | China | *T. aestivum* | KJ536095 |
| WDV-W [HNZD08-3] | China | *T. aestivum* | KJ536127 |
| WDV-W [HNHYK08-4 ] | China | *T. aestivum* | KJ536128 |
| WDV-W [QHXN08-26 ] | China | *H. vulgare* | KJ536148 |
| WDV-W [SXHC10-9] | China | *T. aestivum* | JQ647492 |
| WDV-W [SXHC10-11] | China | *T. aestivum* | JQ647494 |
| WDV-W [SXHC10-14] | China | *T. aestivum* | JQ647496 |
| WDV-W [SXHC10-15] | China | *T. aestivum* | JQ647497 |
| WDV-W [SXHC10-17] | China | *T. aestivum* | JQ647499 |
| WDV-W [SXHC10-18] | China | *T. aestivum* | JQ647500 |
| WDV-B [Iran] | Iran | *H. vulgare* | FJ620684 |
| WDV-B [Bavant] | Iran | *H. vulgare* | JN791096 |
